# New Method of Percussion

**Published:** 1915-02

**Authors:** 


**New Method of Percussion.** A microphone is held before the open mouth of the patient and is connected by means of rubber tubes with the ears of the physician. The latter then percusses gently the various portions of the thorax and can make out accurately the slightest changes in resonance.—Prac. Med., Delhi, Ind.

